# Developing Dynamic Digital Image Techniques with Continuous Parameters to Detect Structural Damage

**DOI:** 10.1155/2013/453468

**Published:** 2013-08-19

**Authors:** Ming-Hsiang Shih, Wen-Pei Sung

**Affiliations:** ^1^Department of Civil Engineering, National Chi Nan University, Nantou 545, Taiwan; ^2^Department of Landscape Architecture, Integrated Research Center for Green Living Technologies, National Chin-Yi University of Technology, Taichung 411, Taiwan

## Abstract

Several earthquakes with strong magnitude occurred globally at various locations, especially the unforgettable tsunami disaster caused by the earthquake in Indonesia and Japan. If the characteristics of structures can be well understood to implement new technology, the damages caused by most natural disasters can be significantly alleviated. In this research, dynamic digital image correlation method for using continuous parameter is applied for developing a low-cost digital image correlation coefficient method with advanced digital cameras and high-speed computers. The experimental study using cantilever test object with defect control confirms that the vibration mode calculated using this proposed method can highly express the defect locations. This proposed method combined with the sensitivity of Inter-Story Drift Mode Shape, IDMS, can also reveal the damage degree of damage structure. These test and analysis results indicate that this proposed method is high enough for applying to achieve the object of real-time online monitoring of structure.

## 1. Introduction

Climate change, caused by global warming, results in the vigorous sea bed volcano activities and plate movement to cause frequent earthquake. Many strong earthquakes, such as Indonesia (2004, 2005), Iran (2005, 2006), Pakistan (2005, 2008), China (2008), Italy and Japan (2009), and New Zealand and Japan (2011), seriously threatened the safety and property of the residents. Particularly, Japan's devastating earthquake and subsequent tsunami, which happened on March 11, 2011, with Richter magnitude scale 9.0, brought about more than sixty thousand casualties and three thousand hundred million US dollar economic loss. Additionally, climate abnormality, caused by greenhouse effect to induce climate change, brings about more frequent typhoon with torrential rains that causes flood, landslide, and river bed erosion that lead to bury the whole village, as what happened in south Taiwan on August, 2009. This incident caused great loss of human life and economic loss and property damages. Taiwan is located on the circum-Pacific seismic belt. The life and property of people living in Taiwan are seriously threatened by the shortening of strong earthquake period and increasing precipitation intensity. Therefore, monitoring the seismic proof capacity of old building and strengthening its structural antiseismic capacity will assist these kinds of buildings in sustaining natural calamities and protect human life. The evaluation method for antiseismic capacity of building must provide early warming, evacuating, and strengthening buildings and allow the comparison of structural function of building before and after damage to make a decision to strengthen or replace building. Hence, the cost-effective and reliable monitoring method, based on developing and applying digital image correlation method, DIC, for using continuous parameter with sensitivity of Inter-Story Drift Mode Shape, IDMS [1, 2], to monitor building deformation and movement, is proposed in this study.

 The digital image correlation method is proposed by Peter et al. [3] in 1982. Then, many scholars applied and inferred mechanics and mathematics theories for expanding the application of DIC method [4–10]. The research team of digital image correlation method, Taiwan, has developed two-dimensional DIC method for observing tiny crack phenomenon cracks in brick walls, warp cracks developed in reinforced concrete, cracks developed in brittle material, and warp cracks developed in light aggregate concrete. It was also applied to observe microscopically metal anisotropic behavior, testing steel plate damages mechanically. Then, this research team developed dynamic DIC method to monitor bridge deformation under traffic loads and the structural dynamic response of reducedscale five-story building under excitation of earthquake forces [10]. These test results achieve quite well-experimental results, compared with the traditional test method. Thus, this research develops this real-time monitoring techniques based on establishing the correlation coefficients of digital images, for using continuous parameter with Inter-Story Drift Mode Shape, IDMS, to analyze cantilever beam with various damage conditions and locations.

## 2. Methodology

### 2.1. Static Digital Image Correlation Method

**Figure 1:**
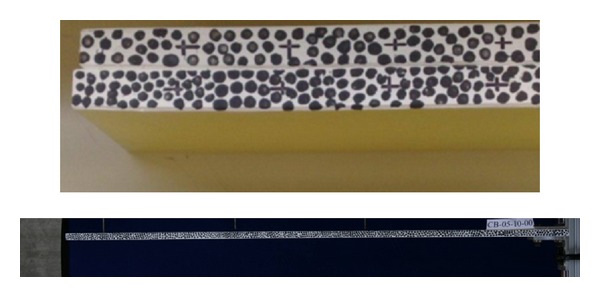
The “structural speckle” marked on the specimen surface of cantilever beam.

**Figure 2:**
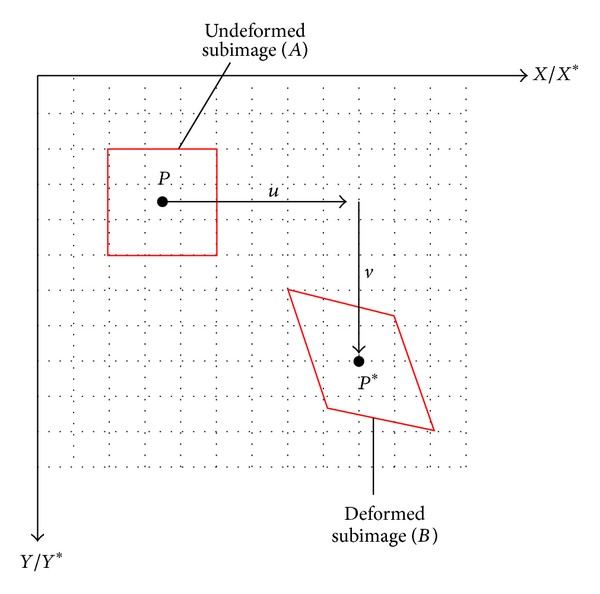
Schematic drawing of relative location of deformed and undeformed subimages.

The principle of digital image correlation method, DIC, is based on the “Finding Algorithm” that developed the technology of DIC for comparing the local correlation of two images before and after deformation. Therefore, the main concept of digital image correlation method is based on the finite element method. The images are divided into small mesh. The mesh of original image can be compared with those in the image after transformation so that the corresponding location of this selected zone in the deformed image can be identified. Thus, “structural speckle” will be established on the specimen surface of cantilever beam, shown in Figure 1. This makes a different grayscale distribution in the image. This grayscale distribution characteristic can be utilized to identify the corresponding position of images before and after displacement. The relationship between deformed and undeformed images can be identified. The central point prior to deformation is point *P*; it is changed to point P* after deformation, shown in Figure 2. The functional relationship is expressed as

(1) $$\begin{array}{c} x^{*}=x+u_{0}+\frac{\partial u}{\partial x}dx+\frac{\partial u}{\partial y}dy, \end{array}$$

(2) $$\begin{array}{c} y^{*}=y+v_{0}+\frac{\partial v}{\partial y}dx+\frac{\partial v}{\partial y}dy. \end{array}$$

For undeformed images, finite element method (FEM) is used to divide the images into several subimages. Assuming the undeformed subimage is *A* and deformed subimage is *B*, the correlation coefficient (3) [3] is used to define the relationship between subimages *A* and *B*. The correlation coefficient will be equal to 1 when the subimage *B* is exactly the subimage *A* after deformation:

(3) $$\begin{array}{c} \text{COF}=\frac{\sum_{}g_{ij}\;{\tilde{g}}_{\bar{ij}}}{\sqrt{\sum_{}g_{ij}^{2}\cdot \sum_{}{\tilde{g}}_{\bar{ij}}^{2}}}, \end{array}$$

where *g*_*ij*_ and ${\tilde{g}}_{\bar{ij}}$ are grayscale of image *A* on coordinate (*i*, *j*) and image *B* on coordinate ($\bar{i}$, $\bar{j}$), respectively. And coordinate ($\bar{i}$, $\bar{j}$) of image *B* corresponds to coordinate (*i*, *j*) of image *A*.

### 2.2. Dynamic Digital Image Correlation Method

**Figure 3:**
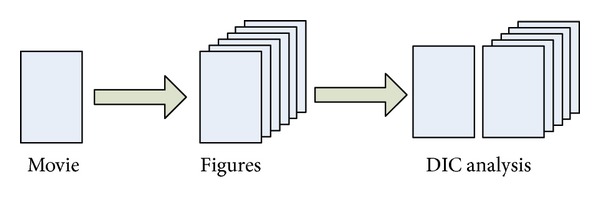
The analysis flowchart of dynamic DIC method.

The dynamic digital image correlation method uses optical equipment to record the process of the object deformation and movement in digital film. Movie can play many photographs quickly and successively, and then persistence of vision of human being is used as visual effect of motion picture. Therefore, the images of movie can be taken apart for analyzing the object deformation and movement by digital image correlation method, shown in Figure 3. 

Otherwise, identifying the dynamic response of cantilever beam must meet requirement of sensitive instruments and equipment with sufficient frequency or sufficient readings for each recoding channel within a unit time. Hence, Camel NexShot 2C-2.1 M, made in Taiwan, CMOS industrial camera with 41 Hz capturing frequency, 1600 × 1200 dots per inch and the highest sampling rate reaches to 15 fps, is used to collect uncompressed photographs with high quality in this study. Because the recording frequency is insufficient, the scanning numbers of selecting images adjust 120 pieces; selecting image reaches to 42 pictures per minute. Then, the collected images can be analyzed for object displacement by the DIC method.

### 2.3. Damage Index for Cantilever Beam

 When the structure of building is damaged, the stiffness of structure descended that reacts to displacement response of structure. Therefore, in order to investigate the relation between structural damage and variation of drift mode of structure, the Inter-Story Drift Mode Shape, IDMS, is used to detect the structural damage in this study. The story drift of this test cantilever beam before damage is used as datum line to compare the damaged story drift of test cantilever beam. IDMS is only considered the first mode of inter-story drift mode to express its sensitivity. In this paper, the first mode variation of structure with and without damage under the excitation of external force to induce structural injury is proposed to be damage index to detect the structural damage. 

The sensitivity of Inter-Story Drift Mode Shape, IDMS, is expressed as follows:

(4) $$\begin{array}{c} S_{i}=\frac{D_{i}-B_{i}}{B_{i}}\times 100\%, \end{array}$$

where *S*_*i*_ represents the sensitivity of story drift mode variation of the first mode at the *i*th position after damage. *B*_*i*_ represents the normalized story drift mode variation of the first mode at the *i*th position before damage. *D*_*i*_ represents the normalized story drift mode variation of the first mode at the *i*th position after damage.

## 3. Experimental Setup

**Figure 4:**
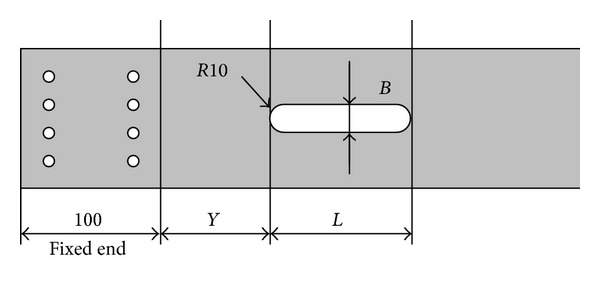
The definition of defects of test sample of this study.

**Figure 5:**
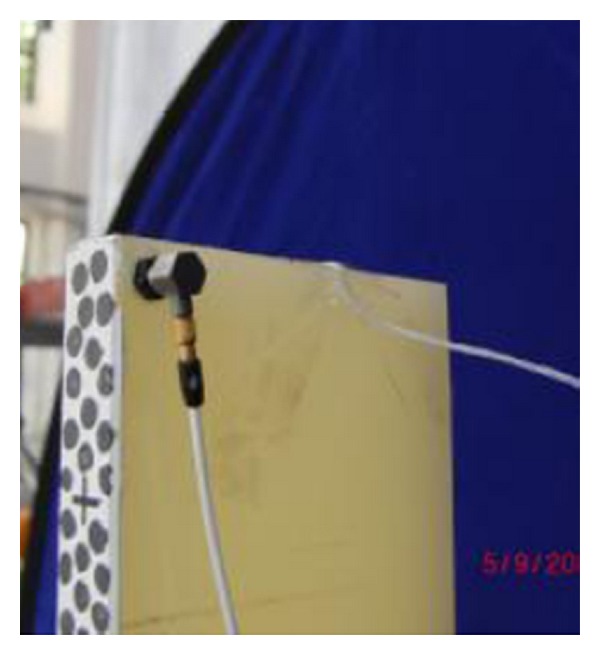
Installed accelerometer at the end of tested sample.

**Figure 6:**
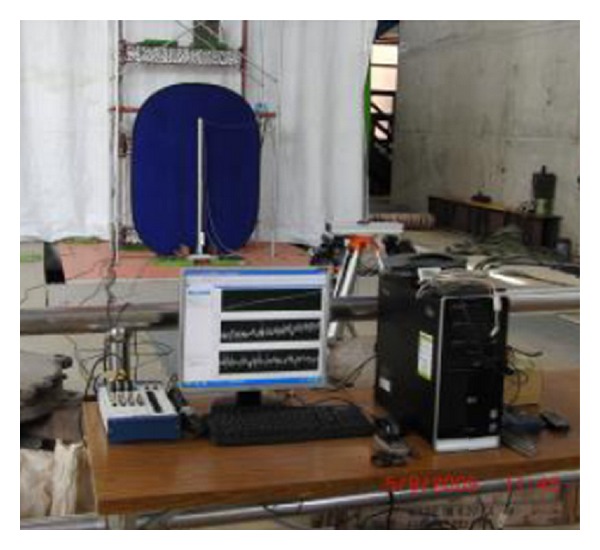
The experimental setup in this study.

The objective of this research is to propose the DIC method to develop continuous parameters to detect static and dynamic deformation and damage evaluation for structure. In order to test and verify the DIC techniques for evaluating the state and dynamic state measurement of cantilever beam, a reducedscale cantilever-vibrating platform is designed to investigate the capability of this proposed continuous parameters method. The characteristics of this test model of reducedscale cantilever-vibrating platform are described as follows: dimensions 1398 mm long, 95 mm wide, and 20 mm thick; material is 20 mm plastic plate with defects, contained various dimensions and locations, made on the central axis of the test object with milling machine by reducing the modulus of the cross-section of the test object at where the defect is located, shown in Figure 4. The structural spot making is painted white and then marked randomly with 4 mm dark-gray spots in the surface. Then, accelerometers were installed at the fixed end, the midpoint, and the free end of the cantilever to record acceleration response at 1000 Hz, shown in Figure 5. The test sample was fixed on the earthquake simulator, subjected to excitation of Kobe earthquake. All dynamic responses were recorded by CMOS industrial camera and high-speed computer. The test setup in this study is shown in Figure 6. The first test sample is original cantilever beam without any defect, serial number CB-00-00-00. Then, the other test cantilever beams with various defects is defined by using Figure 4 to define the serial number. The identification numbers of test samples are listed in Table 1. 

## 4. Experimental Results and Discussions

### 4.1. Test Verification

**Figure 7:**
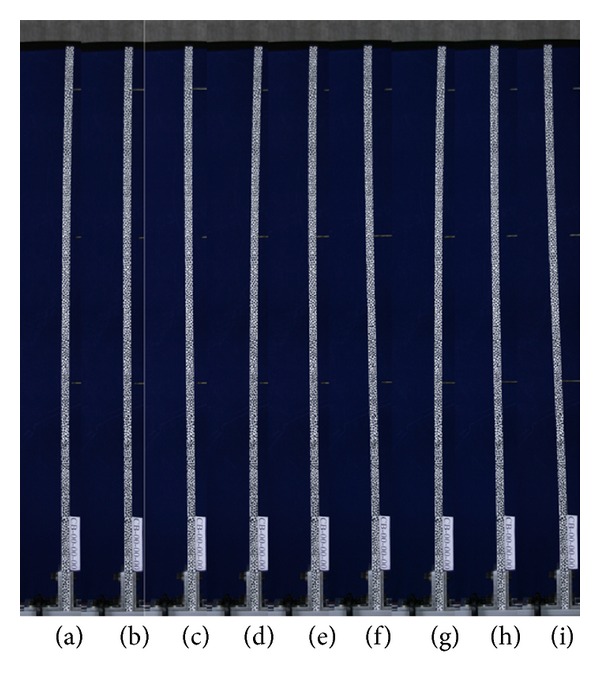
The images are taken apart for analyzing the object deformation and movement.

**Figure 8:**
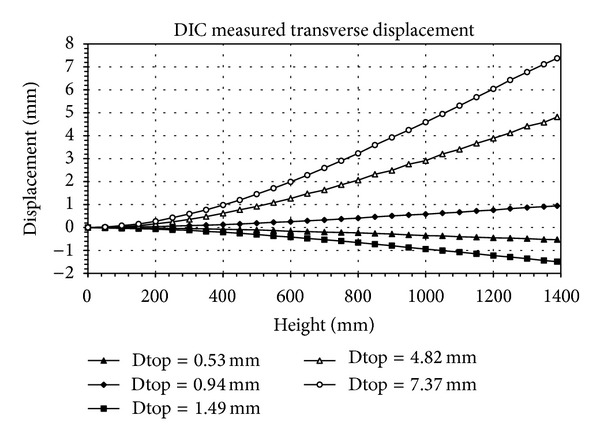
Displacement curve.

**Figure 9:**
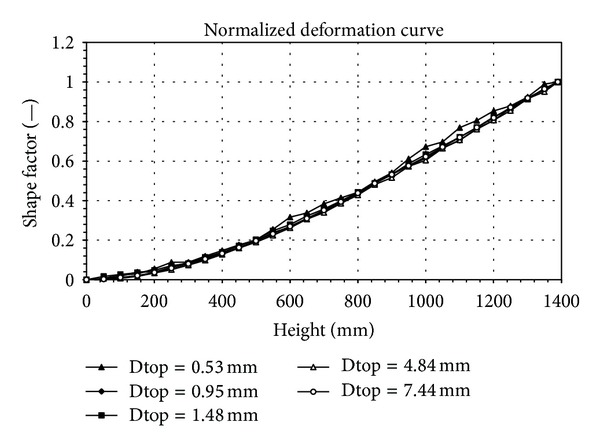
Normalized displacement curve.

In order to verify the accuracy of this proposed method, there are five free-vibration tests and the images are picked from the test, applied to the developed DIC analyzing program. The images records, picked from the test, are taken apart for analyzing the object deformation and movement, as shown in Figure 7. The five analysis displacement curves for dynamic test are shown in Figure 8. The regressive analysis by four polynomials is applied to normalize these five analysis displacement curves. The R-square of these five displacement curve values in order are 0.99882, 0.99971, 0.99982, 0.99986, and 0.99998. The sensitivity for these tests is 0.427 mm/pixel. These five normalized curves have been turned to transform into a curve to fit together regularly, shown in Figure 9, except that the curve of top displacement is 0.53 mm. The deviation of this curve is larger than the other curves. Thus, it is clear to see that its relativity is good. Therefore, these test results can confirm the accuracy of the digital image technique and analyzing program.

### 4.2. Dynamic Test and Structural Damage Evaluation

**Figure 10:**
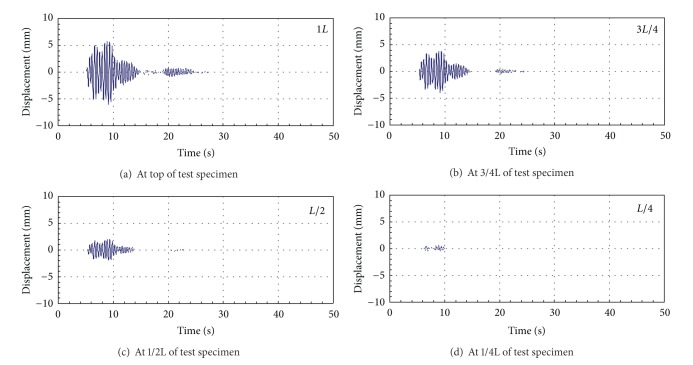
The time history of displacement response of test specimen under excitation of Kobe earthquake at top, 3/4*L*, 1/2*L*, and 1/4*L*.

**Figure 11:**
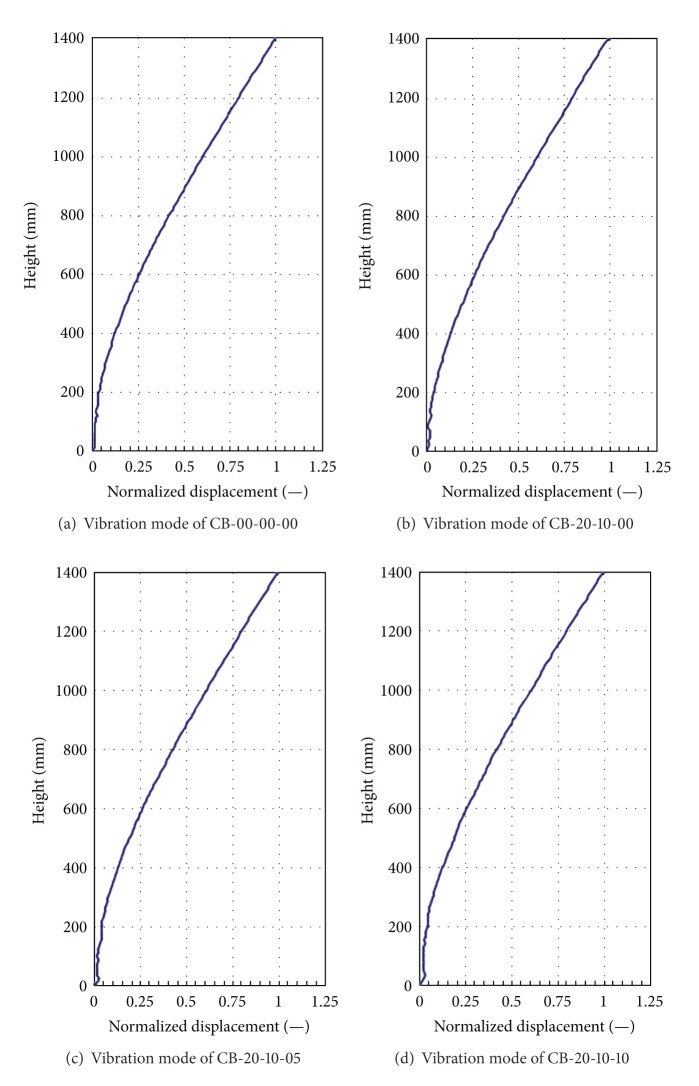
The remarkable vibration mode of various test specimens.

**Figure 12:**
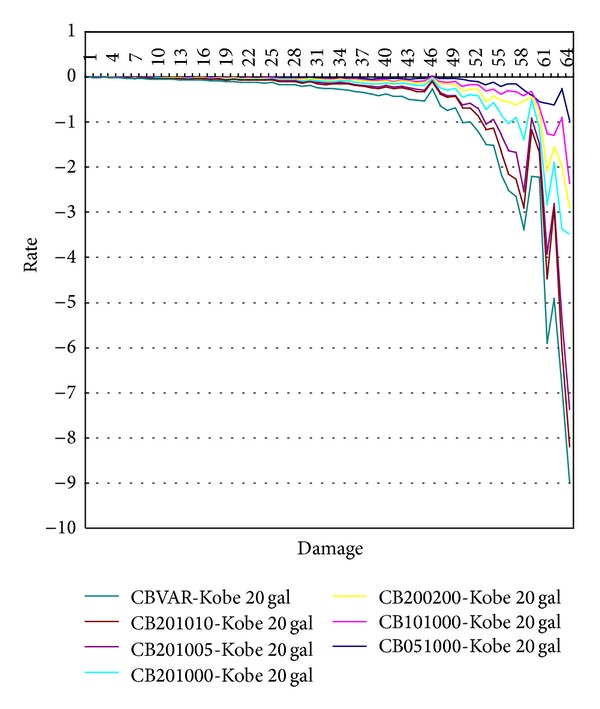
The analysis results of IDMS for various test specimens.

In order to assess the structural damage for using continuous parameters to improve DIC techniques, this reducedscale cantilever beam needs to fabricate defects with various dimensions and locations to investigate the practicability of DIC for using continuous parameters to monitor dynamic response of these test samples.  Figure 10 shows that the time history of displacement response at the end, 3/4 length, 1/2 length, and 1/4 length of test specimen under excitation of Kobe earthquake is recorded, respectively. Then, the selected vibration modes of serial numbers CB-00-00-00, CB-20-10-00, CB-20-10-05, and CB-20-10-10 are shown in Figure 11, respectively, indicating that the vibration mode can easily reflect the location of structural damage. Nevertheless, these vibration modes cannot show the damage situation of these test specimens. Therefore, the sensitivity of Inter-Story Drift Mode Shape, IDMS, is used to evaluate the damage conditions of structure. The analysis results are shown in Figure 12. This figure indicates that this continuous parameter method to improve DIC techniques can easily reflect the damage locations and structural damage conditions. 

## 5. Conclusions 

In order to investigate the practicability of using the continuous parameter to improve the DIC method to detect dynamic response of structural damage under the excitation of external force, reducedscale cantilever beams without defect and with various defects are evaluated for external earthquake force by monitoring of dynamic response with device of Camel NexShot 2C-2.1 M. The analysis results are summarized as follows.

1. The time history of displacement curves for free-vibration experiment, passed through normalization, shows that these normalized curves are tallied with each other and the data recorded from accelerometers approximately. These tests' results demonstrate that the conformability of this digital image technique and analyzing program, proposed in this study, is very good.
2. The test results indicate that the practicability of using the continuous parameter to improve the DIC method to detect dynamic response of structural damage under the excitation of external force can not only acquire the reliable time history of displacement response for dynamic state test but also can achieve correct vibration mode. Particularly, these vibration modes can express the structural damage on these test specimens.
3. This proposed method combined with the sensitivity of Inter-Story Drift Mode Shape, IDMS, not only can express the true damage position of these test specimens but also can reveal the damage degree of damage structure. This proposed method is very useful for monitoring the structural damage.

The test results demonstrate that using the continuous parameter to improve the DIC method to detect dynamic response of structural damage under the excitation of external force has high practicability. All of these test results indicated that the accuracy degree of this proposed method is high enough for applying it to achieve dynamic response of building under excitation of external force.

## Figures and Tables

**Table 1:** The identification number of specimen number.

Specimen No.	*B* mm	*L* cm	*Y* cm
CB-00-00-00	0	0	0
CB-05-10-00	5	10	0
CB-10-10-00	10	10	0
CB-20-10-00	20	10	0
CB-20-02-00	20	2.5	0
CB-20-10-05	20	10	5
CB-20-10-10	20	10	10
